# Pyrvinium Pamoate and Structural Analogs Are Early Macrofilaricide Leads

**DOI:** 10.3390/ph15020189

**Published:** 2022-02-02

**Authors:** Emma L. Gunderson, Clifford Bryant, Christina A. Bulman, Chelsea Fischer, Mona Luo, Ian Vogel, Kee-Chong Lim, Shabnam Jawahar, Nancy Tricoche, Denis Voronin, Christopher Corbo, Rene B. Ayiseh, Faustin P. T. Manfo, Glory E. Mbah, Fidelis Cho-Ngwa, Brenda Beerntsen, Adam R. Renslo, Sara Lustigman, Judy A. Sakanari

**Affiliations:** 1 Department of Pharmaceutical Chemistry, University of California San Francisco, San Francisco, CA 94158, USA; emma.gunderson@ucsf.edu (E.L.G.); clifford_bryant@sbcglobal.net (C.B.); cabulman@dons.usfca.edu (C.A.B.); chelsea.fischer811@gmail.com (C.F.); mona.luo144@gmail.com (M.L.); ian.vogel@irb.usi.ch (I.V.); limitskc@hotmail.com (K.-C.L.); adam.renslo@ucsf.edu (A.R.R.); 2 Molecular Parasitology, New York Blood Center, Lindsley F. Kimball Research Institute, New York, NY 10065, USA; shabnam.mob@gmail.com (S.J.); ntricoche@nybc.org (N.T.); denis.voronin@nih.gov (D.V.); 3 Department of Biological Sciences, Wagner College, Staten Island, NY 10301, USA; ccorbo@wagner.edu; 4 ANDI Centre of Excellence for Onchocerciasis Drug Research, Biotechnology Unit, Faculty of Science, University of Buea, Buea P.O. Box 63, Cameroon; rbilingwe@yahoo.com (R.B.A.); faustinpascal@yahoo.fr (F.P.T.M.); gmbah21@googlemail.com (G.E.M.); chongwa_ub@yahoo.co.uk (F.C.-N.); 5 Higher Teacher Training College (HTTC), The University of Bamenda, Bamenda P.O. Box 39, Cameroon; 6 Department of Veterinary Pathobiology, University of Missouri-Columbia, Columbia, MO 65211, USA; beerntsenb@missouri.edu

**Keywords:** repurposed drugs, pyrvinium pamoate, filarial diseases, macrofilaricides

## Abstract

Onchocerciasis and lymphatic filariasis are neglected tropical diseases caused by infection with filarial worms. Annual or biannual mass drug administration with microfilaricidal drugs that kill the microfilarial stages of the parasites has helped reduce infection rates and thus prevent transmission of both infections. However, success depends on high population coverage that is maintained for the duration of the adult worm’s lifespan. Given that these filarial worms can live up to 14 years in their human hosts, a macrofilaricidal drug would vastly accelerate elimination efforts. Here, we have evaluated the repurposed drug pyrvinium pamoate as well as newly synthesized analogs of pyrvinium for their efficacy against filarial worms in vitro and in vivo. We found that pyrvinium pamoate, tetrahydropyrvinium and one of the analogs were highly potent in inhibiting worms in in vitro whole-worm screening assays, and that all three compounds reduced female worm fecundity and inhibited embryogenesis in the *Brugia pahangi*-gerbil in vivo model of infection.

## 1. Introduction

According to the 2017 Global Burden of Disease Study, over 20 million people worldwide are infected with the filarial worm *Onchocerca volvulus,* which causes onchocerciasis, and nearly 65 million people are infected with *Wuchereria bancrofti*, *Brugia malayi* or *B. timori*, the filarial worms that cause lymphatic filariasis (LF) [1]. Onchocerciasis, also known as river blindness, is a chronic disease caused by the first larval stage, microfilariae (mf), that are released from female worms residing in subcutaneous tissues. The mf migrate throughout the skin, causing severe pruritus, and when they migrate to the eyes, they induce an inflammatory response that eventually leads to impaired vision and blindness. Lymphatic filariasis (elephantiasis) is also a chronic disease but the pathology is associated with the adult stages that induce inflammation that can permanently damage host lymphatic tissues [2]. Symptoms of this disease include chronic pain associated with severe lymphedema typically in the arms, legs, breasts and genitalia. Individuals affected by onchocerciasis and elephantiasis often experience economic hardship, social stigma, and suffering. Together, LF and onchocerciasis cause over 2.7 million years lived with disability (YLDs) to people living in impoverished countries [1].

Significant progress in decreasing the prevalence and transmission of these infections has been made in recent years, largely through annual or biannual mass drug administration (MDA) programs lead by the World Health Organization with drugs that are effective against the microfilarial stage of the parasite [1,3,4]. However, there is still a critical need for safe, effective drugs that kill the adult worms (macrofilaricides) since female worms release millions of mf over their lifespan (6–14 years for these filarial worms) [5], that infect the blackfly (*O. volvulus*) and mosquito (LF) vectors, thus perpetuating the life cycle. Another major issue with the current microfilaricides is that ivermectin (IVM) used in both MDA programs is not recommended for MDA in regions co-endemic with another filarial nematode, *Loa loa*, because individuals with high numbers of *L. loa* mf are at risk of developing severe adverse reactions [6,7]. Moreover, while current evidence for IVM resistance in *O. volvulus* is far from definitive, sub-optimal responses to IVM in the treatment of onchocerciasis have been identified, i.e., faster rates of skin repopulation are probably due to decreased effects of IVM on female worm fecundity. Ideally, a macrofilaricide would be used in MDA programs and/or in test and treat strategies to cure infections or hinder fecundity, thereby stopping mf transmission and thus shortening the time to elimination of these diseases. Presently, the target year for eliminating LF and onchocerciasis is 2030 [8,9,10].

Previously, we screened a library of FDA-approved drugs and identified the anthelminthic drug, pyrvinium pamoate (PVP), as a potent hit in in vitro filarial worm assays [11]. PVP has been used over the last 50 years to treat pinworm infections (enterobiasis) in children; however, it is not readily bioavailable as it is not appreciably absorbed by the gastrointestinal tract [12,13]. Several analogs of PVP were generated in an effort to improve its bioavailability for use in other applications such as treatment for prostate cancer [14,15]. Here, we describe the evaluation of PVP as well as several newly synthesized synthetic analogs in which the quinoline ring was reduced to tetrahydroquinoline, substitution patterns on the pyrrole ring were altered, or the potentially oxidation-prone pyrrole ring was replaced by pyrrazole. The present study identified two new analogs, tetrahydropyrvinium (THP) and pyrvinium analog 06, as the most promising early macrofilaricidal compounds using in vitro phenotypic screens and the *Brugia pahangi*-gerbil model of infection.

## 2. Results

### 2.1. Analog 06 Shows a Favorable Inhibitory Profile across Parasite Species and Life Stages In Vitro

**Figure 1:**
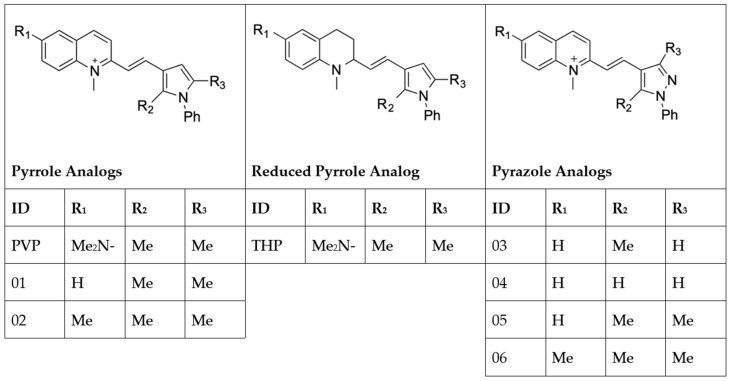
Chemical structures of pyrvinium pamoate and its analogs.

PVP, tetrahydropyrvinium (THP), and six other PVP analogs that were modified at the quinoline or pyrrole rings (Figure 1) were initially screened in a *B. pahangi* adult female motility assay.

*B. pahangi* adult females are readily accessible from laboratory animals and are used in primary screens to test compounds in vitro. Compounds that inhibit motility of adult female *B*. *pahangi* by >70% are further tested with *O*. *ochengi* adults and mf, as well as *L*. *loa* mf; they are then prioritized for screening in the *O*. *volvulus* pre-adult (L5) assays and *O. volvulus* third-stage larvae (L3) molting assay when compounds are effective in the low micromolar range (<5 µM). Six out of the 8 compounds (PVP, THP, 01, 02, 05 and 06) tested with *B. pahangi* inhibited worm motility by >70% and had IC_50_ values in the sub-micromolar range (Table 1). The most potent compound was PVP with an IC_50_ of 0.3 nM.

The same six compounds also showed strong potency against *O. ochengi* adult males and females, with IC_50_s in the low µM or sub-micromolar range. Compounds 04 and 05 inhibited motility of male *O*. *ochengi* by 100% and 86%, respectively, at a single concentration of 10 µM but were deprioritized since 04 was not as potent as the other compounds and 05 had a sub-optimal ADME profile (Table 2).

The microfilariae of *O. ochengi* and *L. loa* were screened with all 8 compounds with single concentrations of 10 µM. PVP, THP, 01 and 02 completely inhibited mf motility, while 03, 04 and 05 had little to no effect on the mf. Analog 06 inhibited *O*. *ochengi* mf motility by 46% and inhibited motility of *L*. *loa* mf by 88% at 10 µM. Because the number of *L*. *loa* mf needed for IC_50_ assays were not readily available, an IC_50_ was only conducted for PVP which had a value of 0.3 µM, a value 3- and 6-fold higher than those for adult male and female *O*. *ochengi* worms, respectively.

**Figure 2:**
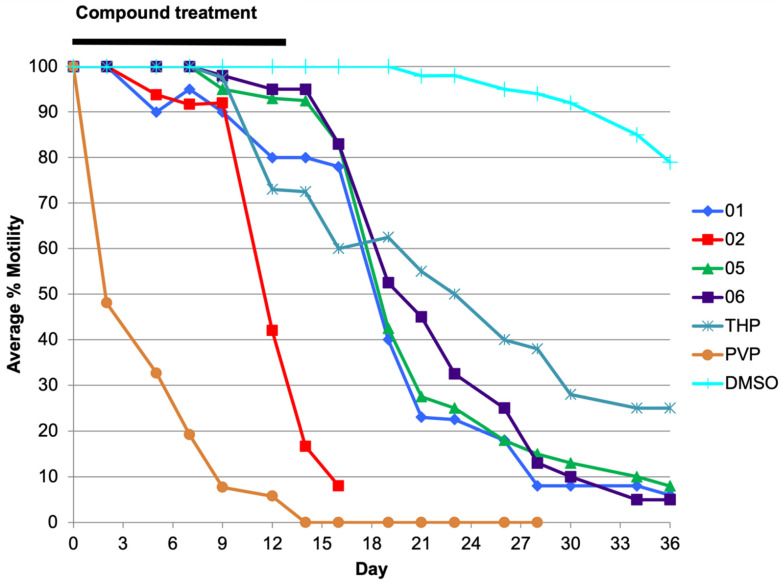
Ov L5 were treated with 1 µM of 01, 02, 05, 06, THP and PVP for 14 days (change of media + drug 50% every two days) and then with fresh media (change of media 50% up to day 36). PVP treated L5 worms did not recover during this washout period and the assay with analog 02 was stopped on day 16 when contamination was observed. Inhibition of motility was well correlated with inhibition of viability as measured by endpoint MTT assay (Supporting Information Table S1).

Analogs 03, 06, THP and PVP were also tested in an *O. volvulus* L3 (Ov L3) molting assay and all four compounds had IC_50_s in the sub-micromolar range (<0.5 µM; Supporting Information Figure S1). Compounds 01, 02, 05, 06, THP and PVP were also tested with *O. volvulus* pre-adult L5 (Ov L5) at 1 µM over 36 days (Table 1, Figure 2). Compounds 01, 02, 05, 06 and PVP were highly effective in inhibiting Ov L5 motility (>90% by day 36) when treated with 1 µM, compared to THP (68% by day 36).

### 2.2. Ov L5 Treated with 10 µM THP In Vitro Show Ultrastructural Damage

**Figure 3:**
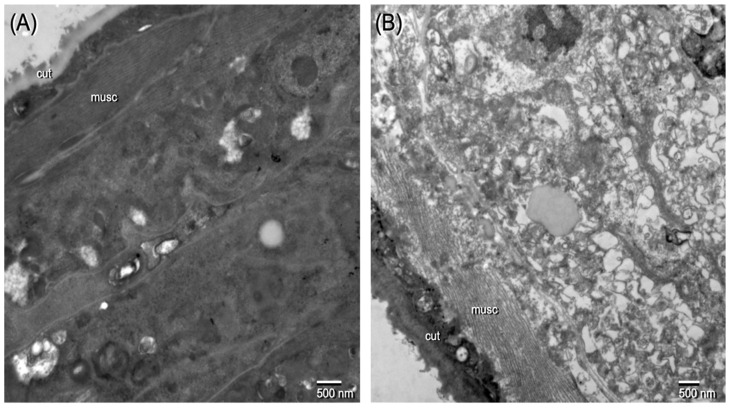
Microphotograph of (**A**) control and (**B**) THP treated Ov L5 from an in vitro assay. THP treated Ov L5 show cellular damage: vacuolization of cytoplasm, disrupted morphology of cellular content and cells lacking integrity. cut = cuticle; musc = muscle.

Ov L5 worms treated with THP in vitro were assessed by transmission electron microscopy (TEM) to examine the qualitative effects of drug treatment on the ultrastructural integrity of the worms. The Ov L5 were cultured in vitro to day 78 and then treated for 14 days with 10 µM THP, exchanging the media with compound every other day. After the 14 days of treatment, the Ov L5 were kept in fresh media for the next 7 days, with media exchanged every other day. THP treatment caused significant damage as evidenced by the significant vacuolization and necrosis (Figure 3) occurring in worms treated with THP.

### 2.3. Analog 06 Demonstrated Acceptable Activity in ADME Studies

Analogs 01, 02, 03, 05, and 06, as well as THP and PVP were tested for stability in gerbil liver microsomes, and permeability across MDCK monolayers, as a model for intestinal absorption (Table 2). Analog 06 showed the most promise in the gerbil liver microsome assay as it was the only compound with low intrinsic clearance (CL_int_ = 0.9 µL/min/mg protein); all other tested compounds had clearance rates ≥95 µL/min/mg. All the compounds exhibited very low permeability in the apical to basal direction (MDCK assay), with significant efflux ratios, indicating a propensity for efflux by the P-gp transporter. Nevertheless, because 06 had the best activity against target worms in vitro and overall, had the most promising ADME profile, it was selected for in vivo studies as well as THP and PVP.

### 2.4. Pyrvinium, THP and 06 Reduce Female Worm Fecundity in the Brugia/Gerbil Model

Animals necropsied 13 weeks post-first dose revealed that adult worm and peritoneal mf numbers were highly reduced (~95%) in animals treated IP once a day for 5 days with 1 mg/kg PVP compared to the control group. Only 6 worms were recovered from animals treated with PVP (*n* = 7 animals; mean = 0.9 worms) while the animals from the vehicle group had a total of 148 worms (*n* = 8 animals; mean = 18.5). Although there was no statistical difference between these two groups due to the high variability within each group, the results suggest that PVP impacts worm burdens since approximately 20-fold fewer worms were recovered from treated animals (Table 3). *Wolbachia* titers were not significantly different in worms from the treated animals compared to the vehicle group, suggesting that compounds had a direct effect on the adult worms and not an indirect effect on the bacteria (Supporting Information Figure S2).

The mean number of mf recovered from control animals was 1.27 × 10^6^, while the mean number of mf from PVP treated animals was 10.4 × 10^3^ mf, which is 100-fold less compared to the control group, suggesting that PVP also greatly reduced female worm fecundity.

**Figure 4:**
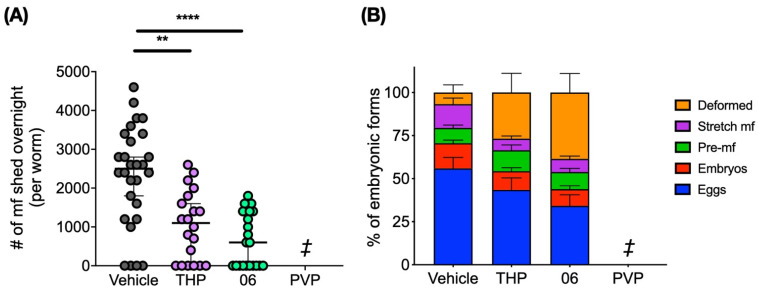
THP and 06 reduce fecundity in female worms recovered from animals treated intraperitoneally with 1 mg/kg. (**A**) Mf released overnight from females recovered from animals treated IP with 1 mg/kg THP and 06 was significantly reduced (**, *p* < 0.01 and ****, *p* < 0.0001, respectively). (**B**) THP and 06 impaired embryogenesis: a minimum of 200 embryonic forms within the ovaries and uteri from female worms were counted and scored based on their developmental stages (eggs, embryos, pre-mf, stretched mf and deformed embryos). There was a statistically significant increase in the number of deformed embryos in the THP group (*p* < 0.05) and 06 group (*p* < 0.001) compared to the vehicle group (Supporting Information Table S2). Data are presented as the mean ± SEM. ‡ Only one female worm was recovered from all the animals treated with 1 mg/kg PVP; therefore, no analyses of overnight mf shed nor embryograms were conducted.

Since only a single female worm was recovered from animals treated with PVP, only female worms from animals treated with THP and 06 were further assessed for the number of mf shed overnight and embryograms, both indicators of reproductive capacity and fecundity. Female worms from the THP and 06 treated groups released significantly lower numbers of mf after 24 h ex vivo (*p* < 0.01 and *p* < 0.0001, respectively) (Figure 4A). Embryogram analyses also revealed that there was a significant increase in the number of deformed embryos present in the gonads of female worms recovered from animals treated with THP (*p* < 0.05) and 06 (*p* < 0.001) compared to the vehicle group (Figure 4B).

### 2.5. THP and Analog 06 Cause Damage to Female Reproductive Structures

**Figure 5:**
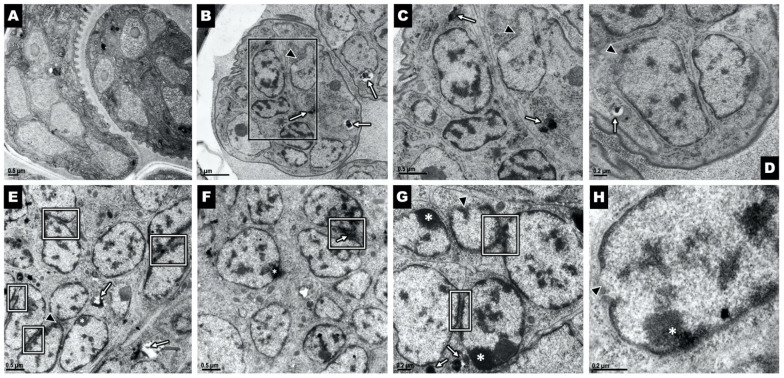
Ultrastructural analysis shows sub-cellular abnormalities in the gonads of the THP- and analog 06-treated worms. (**A**) Microphotograph of a female gonad from a control animal treated with vehicle alone. Cellular inclusions are rare and all nuclear envelopes appear intact. Images (**B**–**D**) are representative images of developing microfilariae inside the reproductive structures of female worms removed from animals treated with THP. The black arrowheads show disrupted nuclear envelopes while white arrows point to internal cellular aggregates. The black box in image B delineates the region seen at a higher magnification in image (**C**). Images (**E**–**H**) are representative images of developing microfilariae inside the gonad of female worms removed from animals treated with analog 06. The white boxes show nuclear membranes that are closely juxtaposed with one another, an unusual feature that may be due to the disruption of the main cellular membrane. White arrows point to aggregates within the cellular structures, which frequently occur at the surface of the nuclear envelope. Arrowheads point to disruption of the nuclear envelope which appears more advanced in worms treated with 06 compared to those treated with THP. The asterisks indicate abnormal accumulation of heterochromatin at the internal surface of the nuclear envelope.

Ultrastructural cell damage was assessed in female worms removed from animals treated with 1 mg/kg of THP and analog 06 using TEM (only 1 female worm was recovered from animals treated with PVP and was not used for TEM). Results support the embryogram analyses and revealed that the ultrastructure of gonads from worms taken from animals treated with THP and 06 contained developing microfilariae that had abnormal ultrastructural phenotypes compared to the controls (Figure 5). Analog 06 (Figure 5E–H) not only had a more severe effect on the gonads but additional cellular pathologies not seen in the THP-treated worms (Figure 5B–D) were also observed, including abnormal heterochromatin accumulation at the inner surface of the nuclear envelope (Figure 5G, white asterisks). Additionally, nuclear envelopes are juxtaposed to each other (Figure 5E–G, white boxes), with the interchromatin contents of each appearing in close proximity to each other. These changes suggest that breakdown of the nuclear membranes within the microfilariae may lead to the disruption of the nuclear envelope as seen in the higher magnification image (Figure 5H, black arrowhead).

## 3. Discussion

Currently, there is no anti-filarial drug that directly targets adult worms nor is there a vaccine available that prevents filarial infection. One strategy to reduce the cost of drug-discovery efforts and accelerate the development of new therapies to treat neglected parasitic diseases is to evaluate repurposed drugs, i.e., those that have already been tested and found to be safe in humans for other indications.

Pyrvinium pamoate (PVP) is an anthelmintic originally used to treat pinworm infections (enterobiasis, oxyuriasis) and strongyloidiasis [16,17,18]. It has also been used in combination with thiabendazole to treat children infected with the parasitic worms *Trichuris trichiura* and *Ascaris lumbricoides* [19]. PVP is also efficacious as an anti-protozoal drug against *Cryptosporidium parvum* [20], *Entamoeba histolytica* [21], *Giardia intestinalis* [21] and *Plasmodium falciparum* [22], and it has been shown to suppress the growth of pathogenic yeast such as *Exophiala dermatitidis* [23] and *Candida albicans* strain i(5L) [24].

To identify repurposed drugs for use as anti-filarial agents, we first screened a library of FDA-approved drugs with adult *B*. *pahangi* in vitro and found that PVP was the most potent compound among the 2200 that were tested; it reduced motility of *B*. *pahangi* with an IC_50_ in the sub-nanomolar range (0.3 nM) [11]. This study extends the initial observation as well as presents the comparative outcomes of testing PVP and several of its analogs on *Brugia* and other filarial parasites. We found that these compounds were highly potent in vitro against filarial worms across species and life stages, with IC_50_s in the sub-micromolar range for all worm types tested.

We also observed that PVP, THP and analog 06 were not only potent against the adult worms but they were also potent in preventing the molting of the infectious stage of *O*. *volvulus* (L3, third-stage larvae). Due to the high cost of distribution of microfilaricides such as ivermectin, mass drug distribution is not implemented in hypo-endemic areas (low prevalence of infections) in Africa, which possibly contributes to continued transmission through human migrations, consequently reintroducing the parasite back into regions where it might have once been controlled [8]. Thus, PVP and its analogs, which may also prevent the infectious L3 from further development during newly acquired infections, show promise as chemoprophylactic drugs [25] as well as potential macrofilaricidal drugs.

Currently, there are test and treat programs in areas co-endemic for onchocerciasis and loaiasis where ivermectin can still be administered through MDA [26]. Epidemiological and modelling studies, however, have indicated that annual ivermectin MDA may not be sufficient to lead to elimination of transmission in previously hyperendemic and holoendemic foci. Moreover, some of the macrofilaricidal candidate drugs in clinical trials performed by DNDi [27] also have some effect on mf but this does not detract from testing them due to the urgent need to identify new macrofilaricidal drugs that can support elimination.

To assess the potential use of PVP, THP and 06 in countries co-endemic for *Loa loa*, further in vitro studies are necessary to determine the IC_50_s of these compounds with mf, as well as validating the potency of these compounds on the mf in animal models [28]. In the present study, in vitro assays were conducted at single concentrations (10 µM) in which PVP, THP and 06 inhibited motility of mf by 100%, 100% and 88%, respectively. The IC_50_ of PVP with *O*. *ochengi* mf was 3- and 6-fold higher than the IC_50_s for adult male and female *O*. *ochengi*, respectively, which suggests that PVP may not be as potent on the microfilarial stage, and thus bodes well for its possible use as a macrofilaricidal drug in countries endemic for both onchocerciasis and loaiasis.

PVP, THP and analog 06 were also assessed in the *B*. *pahangi*-gerbil model of infection. The PVP group had a 95% decrease in worm burden and a 100-fold reduction in the number of microfilariae compared to the vehicle group. Although this was not statistically significant due to high variance, results showed that female worm fecundity was clearly reduced in both the THP and 06 groups. Embryogram analyses showed that both THP and 06 impacted embryogenesis and the reproductive output of the female worms. Moreover, they caused an increase in the number of deformed embryos and a reduction in the number of mf shed overnight compared to the controls. Because transmission of filariasis and the clinical pathology of river blindness is driven by mf, a macrofilaricidal drug that confers long-term sterility of female worms is also viewed as a viable therapeutic approach [29].

Interestingly, the levels of the endosymbiont, *Wolbachia*, were not reduced in any treatment group, suggesting that the effects of the compounds on embryogenesis and fecundity are not mediated through the host-endosymbiont interaction as seen with antibiotics such as doxycycline and rifampicin [29,30,31,32,33,34,35,36,37,38,39], but probably by acting directly on yet unknown targets within the worms that are essential for embryogenesis.

The underlying mechanisms by which PVP, THP and 06 affect adult and larval filarial worms are unknown. In addition to its other anti-parasitic activities, studies have shown that PVP inhibits the proliferation of tumor cells across a range of in vitro and in vivo models of various types of cancers, including colon, breast, lung, prostate, pancreatic cancer, myeloma and lymphoma and has also shown potential as an anti-tuberculosis agent [13,40,41,42,43]. Potential targets of PVP include the Wnt signaling pathway, the Hedgehog pathway, androgen receptors, mitochondrial respiration and glycolysis and further study is needed to determine if PVP and its analogs target similar pathways in filarial worms. It is possible that the application of these compounds, may not only be limited to filarial worm infections but also against other diseases, thus adding value to these new analogs that were synthesized based on the PVP scaffold.

## 4. Materials and Methods

### 4.1. Ethics Statement

All animal experiments were carried out under protocols approved by the University of California, San Francisco Institutional Animal Care and Use Committee (IACUC), approval number AN173847-02, and adhered to the guidelines set forth in the NIH Guide for the Care and Use of Laboratory Animals and the USDA Animal Care Policies.

### 4.2. Synthesis of Pyrvinium Pamoate Analogs

Tetrahydropyrvinium (THP, 2-[2-(2,5-dimethyl-1-phenylpyrrol-3-yl)ethenyl]-N,N,1-trimethyl-2H-quinolin-6-amine) was synthesized as described in US Patent 8,580,773 [15]. Briefly, pyrvinium pamoate (456 mg, 0.4 mmol, 1.0 equiv.) was purchased from US Pharmacopeia (catalog # 1592001) and suspended in ethanol (9 mL), in a 20 mL vial. The mixture was stirred at room temperature under air and sodium borohydride (76 mg, 2.0 mmol, 5.1 equiv.) was added. The vial was capped and the reaction mixture was stirred for 4.5 h, after which time water (2 mL) was added and the resulting suspension was poured into a 25 mL separatory funnel and extracted 3 times with 7 mL dichloromethane. The combined organic layer was dried with sodium sulfate, filtered and concentrated. The residue was purified using automated silica gel chromatography on a 12 g Silicycle cartridge that had been preequilibrated with hexane (Hex) then in a gradient of 0–25% ethyl acetate (EA) in Hex and then 25% EA/Hex. The relevant fractions were collected and concentrated to afford tetrahydropyrvinium (116 mg, 32.2%).

^1^H NMR (300 MHz, chloroform-d) δ ppm 1.51–1.82 (m, 1H), 1.86–2.27 (m, 9H), 2.61–3.09 (m, 11H), 3.78 (brs., 1H), 5.87 (dd, J = 15.6, 7.9 Hz, 1H), 6.18 (s, 1H), 6.45 (d, J = 15.6 Hz, 1H), 6.63 (d, J = 7.9 Hz, 2H), 6.68–6.76 (m, 1H), 7.14–7.24 (m, 2H), 7.36–7.54 (m, 3H). ^13^C NMR (100 MHz, chloroform-d) δ ppm 10.7, 12.7, 25.5, 29.3, 37.5, 42.5, 62.4, 103.1, 112.1, 113.9, 116.1, 117.5, 123.1, 124.0, 125.8, 127.0, 127.8, 128.2, 129.1, 129.6, 138.5, 139.4, 142.7.

LC–MS (ESI) *m*/*z* 386.5 (M+H)^+^.

**Figure 6:**
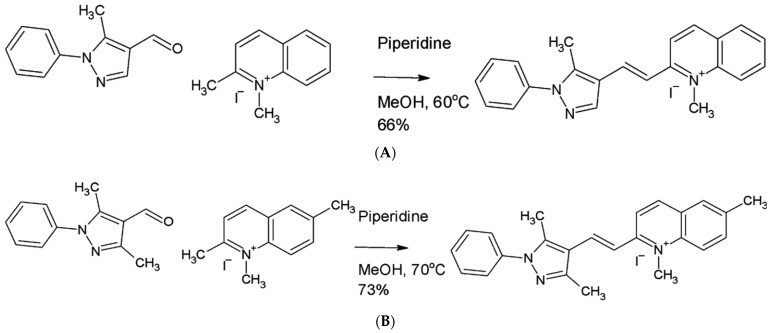
(**A**) Synthesis of analog 03. (**B**) Synthesis of analog 06.

Analogs 01 (2-[(E)-2-(2,5-dimethyl-1-phenyl-1H-pyrrol-3-yl)ethenyl]-1-methylquinolin-1-ium iodide), 02 (2-[(E)-2-(2,5-dimethyl-1-phenyl-1H-pyrrol-3-yl)ethenyl]-1,6-dimethylquinolin-1-ium iodide), 04 (1-methyl-2-[(E)-2-(1-phenyl-1H-pyrazol-4-yl)ethenyl]quinolin-1-ium iodide) and 05 (2-[(E)-2-(3,5-dimethyl-1-phenyl-1H-pyrazol-4-yl)ethenyl]-1-methylquinolin-1-ium iodide) were kindly provided by Dr. Marc Diamond. Analog 03 (1-methyl-2-[(E)-2-(5-methyl-1-phenyl-1H-pyrazol-4-yl)ethenyl]quinolin-1-ium iodide) (Figure 6A) was synthesized as follows. 5-methyl-1-phenyl-1H-pyrazole-4-carbaldehyde (196 mg, 1.05 mmol) and 1,2-dimethylquinolin-1-ium iodide (250 mg, 0.877 mmol) were dissolved and stirred in dry methanol (5 mL) in a septum-capped vial and then purged with dry argon. Piperidine (0.104 mL, 1.05 mmol) was added with a syringe and the reaction was stirred in an aluminum heating block at 60 °C for 4 h, until the reaction was complete as judged by LC–MS. Solvent was removed with a rotary evaporator, and the residue was re-suspended in 12 mL 2:1 (v:v) diethyl ether:methanol and filtered on paper. The filter cake was washed with an additional 6 mL portion of the 2:1 mixture, then diethyl ether, 6 mL. The product was dried in air under foil to protect it from light for 18 h to afford the product (264 mg, 66.4%).

^1^H NMR (400 MHz, DMSO-d6) δ ppm 2.44–2.56 (m, 3H), 4.44 (s, 3H), 7.42 (td, J = 5.8, 2.6 Hz, 1H), 7.46–7.55 (m, 4H), 7.61 (d, J = 15.6 Hz, 1H), 7.82 (t, J = 7.6 Hz, 1H), 8.05 (t, J = 7.5 Hz, 1H), 8.14 (d, J = 15.3 Hz, 1H), 8.23 (d, J = 7.7 Hz, 1H), 8.43 (d, J = 9.0 Hz, 1H), 8.54 (s, 1H), 8.64 (d, J = 9.3 Hz, 1H), 8.89 (d, J = 9.0 Hz, 1H). ^13^C NMR (100 MHz, DMSO-d6) δ ppm 10.8, 39.6, 115.6, 118.8, 119.2, 120.6, 124.8, 127.3, 128.4, 128.5, 129.3, 129.9, 134.5, 138.6, 138.6, 139.1, 139.7, 141.9, 142.9, 156.2.

LC–MS (ESI) *m*/*z* 326.03 (M)^+^.

Analog 06 (2-[(E)-2-(3,5-dimethyl-1-phenyl-1H-pyrazol-4-yl)ethenyl]-1,6-dimethylquinolin-1-ium iodide) (Figure 6B) was synthesized using 3,5-dimethyl-1-phenyl-1H-pyrazole-4-carbaldehyde (175 mg, 0.877 mmol) and 1,2,6-trimethylquinolin-1-ium iodide (250 mg, 0.836 mmol) that were dissolved and stirred in dry methanol (5 mL) in a septum-capped vial and then purged with dry argon. Piperidine (0.120 mL, 1.22 mmol) was added with a syringe and the reaction was stirred in an aluminum heating block at 70 °C for 4 h, until the reaction was complete as judged by LC–MS. Solvent was removed with a rotary evaporator, and the residue was re-suspended in 12 mL 2:1 (v:v) diethyl ether:methanol and filtered on paper. The filter cake was washed with an additional 6 mL portion of the 2:1 mixture, then 6 mL diethyl ether. The product was dried in air under foil to protect from light for 18 h to afford the product (293 mg, 72.8%).

^1^H NMR (400 MHz, DMSO-d6) δ ppm 8.89 (d, J = 9.0 Hz, 1H), 8.64 (d, J = 9.3 Hz, 1H), 8.45 (d, J = 9.3 Hz, 1H), 8.06–8.15 (m, 2H), 8.01 (dd, J = 9.3, 2.0 Hz, 1H), 7.53–7.62 (m, 4H), 7.51 (dd, J = 6.1, 2.4 Hz, 1H), 7.26 (d, J = 16.1 Hz, 1H), 4.49 (s, 3H), 2.54–2.62 (m, 9H). ^13^C NMR (100 MHz, DMSO-d6) δ ppm 11.5, 14.1, 20.6, 39.5, 39.6, 115.4, 116.0, 119.0, 120.7, 124.9, 127.4, 128.3, 128.8, 129.3, 136.4, 137.5, 138.3, 138.4, 138.8, 142.5, 142.6, 149.0, 155.6.

LC–MS (ESI) *m*/*z* 354.07 (M)^+^.

### 4.3. In Vitro Motility Assays with Adult Female Brugia pahangi

A series of in vitro assays were conducted to test the activity of pyrvinium pamoate (PVP), tetrahydropyrvinium (THP), and 6 additional PVP derivatives (01, 02, 03, 04, 05 and 06) with adult female *B*. *pahangi*, adult and mf of *Onchocerca ochengi*, *O*. *volvulus* L3 and L5 pre-adults, and *Loa loa* mf. Compounds were initially screened against *B*. *pahangi* adult females for 3 days in a motility-based assay as previously described [44,45]. Briefly, individual *B*. *pahangi* females were plated into each well of a 24-well plate containing 0.5 mL *Brugia* culture media (RPMI-1640 with 25 mM HEPES, 2.0 g/L NaHCO_3_, 5% heat inactivated fetal bovine serum (FBS), and 1× Antibiotic/Antimycotic solution), then treated with 1 µM compound; media containing 1% DMSO was used as negative controls. Worms were kept in a 37 °C incubator with 5% CO_2_. Motility was monitored daily using the Worminator system [44] and percent inhibition was calculated as compared to the mean movement units (MMUs) of 1% DMSO controls. Compounds that reduced worm motility by 70% or greater were tested using a 6-point serial dilution of compound (ranging from 30 to 1 × 10^−5^ µM), and IC_50_s were calculated using GraphPad Prism software.

### 4.4. In Vitro Assays with Adult Onchocerca ochengi and Mf and Loa loa Mf

As humans are the only hosts for *O*. *volvulus* which are unavailable for screening, drug discovery relies on the surrogate filarial parasites *Brugia spp*. and cattle *Onchocerca spp*. Prioritized compounds from the *Brugia* worm screens were further tested with female and male adult *O*. *ochengi* as well as *O*. *ochengi* and *L*. *loa* mf. IC_50_s were calculated for assays performed with female and male adults for all compounds except for 04 and 05, which were assayed with *O*. *ochengi* males at 10 µM only.

For these assays, *O*. *ochengi* adults were extracted from the subcutaneous nodules of infected cow skins purchased from the local slaughterhouse (Douala, Littoral Region, Cameroon) and incubated in 4 mL complete culture media comprised of RPMI-1640 (Sigma-Aldrich), 5% newborn calf serum, 200 units/mL penicillin, 200 μg/mL streptomycin and 2.5 μg/mL amphotericin B (Sigma-Aldrich) in 12-well plates overnight to validate sterility prior to the assays [44]. Four to eight *O*. *ochengi* adult masses were treated individually the following day with a serial dilution of each compound, and controls were treated with 1% DMSO. While in culture, the male worms normally egress from the nodules into the surrounding media and thus can be monitored in parallel for motility. The *O*. *ochengi* adult masses were incubated at 37 °C with 5% CO_2_ for the duration of the assay. Male motility within each well was visually scored every day and over 5 days after compound addition, and female viability within the nodules was assessed by MTT after 7 days as previously described [44]. The *O*. *ochengi* adult masses containing female worms were individually placed in wells of a 48-well plate with 0.5 mL of 0.5 mg/mL MTT in PBS and incubated at 37 °C for 30 min. Female viability was visually rated by the degree of formazan production (live worms have blue coloration) in the female worm within the treated nodule versus control worm masses. To calculate the IC_50_, quadruplicate worm masses were incubated with final concentrations of 30, 10, 3, 1, 0.3, 0.1 and 0.03 μM and assays were conducted as described above. Prism 4.0 for Windows was used to calculate IC_50_s.

To assess the effects of the compounds on *O*. *ochengi* mf, the mf were harvested from infected cow skin and cultured (10–15 mf per well) in 100 µL media in 96-well plates with a confluent feeder layer of monkey kidney epithelial cells (MK2) as previously described [44]. The motility was visually assessed every day and scored on a scale from 0–100% inhibition of motility after 5 days of treatment. IC_50_ for PVP was calculated using GraphPad Prism software. All compounds were also counter-screened with *L*. *loa* mf collected from infected individuals with approval from the University of Buea (IRB approval # 964-19). Mf were separated from blood cells using a Percoll gradient as previously described [44] and set up for assays (10–15 mf per well). Treated (10 µM of compound) mf were incubated at 37 °C and 5% CO_2_, and the motility was visually assessed every day and finally scored for 0–100% inhibition of motility after 5 days of treatment.

### 4.5. Onchocerca volvulus Molting Assay of Third-Stage Larvae (L3) to Fourth-Stage Larvae (L4)

*O*. *volvulus*, like other nematodes, undergo molts during their larval stages as they develop into the adult form. If the larval worms do not undergo the molt and their outer cuticle is partially or not shed, they will not survive. The molting assay tests the potency of compounds to prevent the L3 from molting to the L4 stage in vitro. PVP, THP and analogs 03 and 06 were tested in an *O*. *volvulus* L3 molting assay as previously described [44]. Briefly, 5–10 Ov L3 collected from infected *Simulium* blackflies and frozen in liquid nitrogen were thawed and plated in 96-well plates seeded with 0.2 × 10^6^ normal human peripheral blood mononuclear cells (PBMCs) in 100 µL complete medium containing 20% heat inactivated FBS and treated with compounds at 10, 3, 1, 0.3, 0.1 and 0.03 μM. L3 exposed to 0.5% DMSO or complete media only were run as negative controls. Larvae were incubated at 37 °C with 5% CO_2_, and molting was determined by examining the wells for highly motile L4 larvae and empty L3 casts after 6 days in culture. Inhibition of molting was calculated relative to DMSO controls, and IC_50_s were calculated using GraphPad Prism software.

### 4.6. Onchocerca volvulus Pre-Adult (Ov L5) Assay

*O*. *volvulus* adult male and female worms, which can only be obtained from infected individuals, are not readily accessible for ex vivo studies. Moreover, since there is no small animal model that can maintain infections by *O*. *volvulus*, a recently developed in vitro system was used to evaluate the macrofilaricidal effects on pre-adult worms [45]. All compounds except for 04 were tested in this *O*. *volvulus* pre-adult/L5 motility and viability assay.

Briefly, Ov L3 larvae were cultured to the pre-adult/L5 stage in vitro as described by Voronin et al., 2019 [45]. Once larvae reached the L5 stage (after at least 70 days in culture), 8–10 Ov L5 were moved to new transwell inserts in a 24-well plate containing 0.5 mL Ov L4-CMS media seeded with a 3 × 10^4^/well Human Umbilical Vein Endothelial Cells (HUVEC) monolayer. Duplicate wells were treated with 1 and/or 10 µM of compound. Ov L5 were exposed to compounds for 14 days (exchanged 50% of media with fresh compound every two days) and then cultured with new media without compound, exchanging 50% of media every 2–3 days, up to day 36. Control Ov L5 were treated with 0.05% DMSO. Concentrations were chosen based on activity in the *B*. *pahangi* female motility assay. Motility was assessed visually and ranked on a 0–100% scale every 2–3 days over the course of the assay. At the end of the motility assay, Ov L5 viability was measured by 3-(4,5-dimethylthiazol-2-yl)-2,5-diphenyltetrazolium bromide (MTT) assay. Ov L5 were incubated with 0.1% MTT in PBS at 37 °C for 1 h then visually inspected for blue coloration indicating MTT reduction. Worms were considered dead if <50% of the worm was stained blue, and worms were considered alive if >50% of the worm was stained blue.

### 4.7. In Vitro Absorption, Distribution, Metabolism and Excretion (ADME)

In vitro gerbil liver microsome assays and MDCK permeability assays were conducted by Quintara Biosciences (South San Francisco, CA) with PVP, THP, 01, 02, 03, 05 and 06 to determine the stability and permeability of each compound. Microsomal stability was tested using 1 µM of each test compound in a 96-well plate containing 0.5 mg/mL gerbil liver microsomes, 1 mM NADPH and/or UDPGA with alamethicin in a buffer of 100 mM potassium phosphate with 3 mM magnesium chloride at pH 7.4. Verapamil was used as a positive control. Reactions were incubated at 37 °C, and 100% acetonitrile with 0.1% formic acid was added at 0, 15, 30 and 60 min timepoints to stop the reaction. Samples were then separated over a Waters Atlantis T3 dC18 reverse phase HPLC column with 0.1% formic acid in water and 0.1% formic acid in acetonitrile. Samples were analyzed using an AB Sciex API 4000 LC–MS/MS.

Permeability assays were conducted using 5 µM of each test compound with digoxin as a control. Compounds were added to the apical and basal sides of wells with a confluent layer of MDCK-MDR1 cells that had been pre-washed in Hank’s Balanced Salt Solution with 5 mM HEPES. Transendothelial electrical resistance was used to verify the integrity of the MDCK-MDR1 monolayer. After compound addition, plates were incubated at 37 °C with 5% CO_2_ for 1 h. Samples were analyzed by LC–MS/MS and net flux ratio was calculated using basal-to-apical and apical-to-basal permeability.

### 4.8. In Vivo Studies with the Brugia pahangi/Gerbil Model of Infection

Male Mongolian gerbils 50–60 g, 5–7 weeks in age (*Meriones unguiculatus*, Charles River Laboratories International, Inc., Wilmington, MA, USA) were injected intraperitoneally (IP) with 200 third-larval stage *B*. *pahangi* and treated a minimum of 3 months later when larvae developed into adult worms [46].

PVP, THP, and 06 were dissolved in DMSO (20 mg/mL) and the stock was added to 44% beta-hydroxypropylcyclodextrin (20% solution) and 55% PEG400 for a final compound concentration of 0.2 mg/mL in 1% DMSO. Animals were given IP doses of 1 mg/kg once a day for 5 days based on in vivo studies by Jones et al., 2009 [14] and gerbil PK analyses. Compounds are light sensitive and were shielded from light during formulation. Animals were necropsied 13 weeks post-first dose, and peritoneal cavities were washed with 100 mL of PBS to collect adult worms and mf that had been released from female worms. Adult worms were separated by sex, counted, and processed for subsequent analyses. Mf from the peritoneal cavities were quantified by mixing peritoneal wash 9:1 (*v*/*v*) with 0.04% methylene blue:water and counted using an inverted microscope.

### 4.9. Ex Vivo Microfilarial Release by Female Brugia pahangi Worms and Embryograms

To test the effects of the compounds on female worm fertility, all surviving female worms were examined ex vivo to quantify the amount of mf released and profile the various stages of embryonic development within the uteri of individual female worms. After necropsy, female worms were incubated in individual wells of a 24-well plate with 2 mL *Brugia* culture media at 37 °C and 5% CO_2_. After 24 h, the number of mf released by each adult female was determined by counting the mf in a 0.5 mL sample of the media and multiplying by 4 to calculate the total number of mf. Additionally, embryogram analyses on the individual female worms was performed as described [46]. Briefly, each female worm was homogenized in 500 μL of PBS to release the uterine content. The embryonic developmental stages (eggs, embryos, pre-mf and mf) were determined in two 10 μL aliquots using a compound microscope and hemocytometer. A minimum of 200 events were assessed from each female worm and at least 3 females were analyzed per animal from at least 3 animals per treatment group. The intra-uterine embryogram was expressed as the relative proportions of different stages of development: eggs, embryos, pre-mf, stretched mf and deformed embryos in the total number of events.

### 4.10. qPCR of Wolbachia and Worm DNA in Treated Female Worms

During necropsy, adult worms were collected and frozen in a dry-ice and ethanol bath, then stored at −80 °C. Genomic DNA was extracted using a DNEasy Blood and Tissue Kit (QIAGEN). Female worms were processed individually, and male worms were pooled in groups of 4 per sample for processing. DNA was quantified using a NanoDrop One^c^ (Thermo Fisher Scientific) before conducting qPCR with Genecoepia 2× All-in-One Master Mix (Cat #QP001-01) in a Bio-Rad CFX Connect RT-PCR thermocycler. The single copy genes, *Wolbachia* surface protein (*wsp*) and *Brugia* glutathione S-transferase (*gst*), were used to calculate *wsp:gst* ratios to normalize *Wolbachia* titers to *Brugia* DNA as previously described [46]. The following primers were used to amplify *wsp*, *wsp*_fwd 5′-CCCTGCAAAGGCACAAGTTATTG-3′; *wsp*_rev 5′-CGAGCTCCAGCAAAGAGTTTAATTT-3′ with the following amplification cycles: heating at 95 °C for 15 min, followed by 40 cycles of denaturation at 94 °C for 10 s, annealing at 55 °C for 20 s and elongation at 72 °C for 15 s. The following primers were used to amplify *gst*, *gst*_fwd 5′-GAGACACCTTGCTCGCAAAC-3′; *gst*_rev 5′-ATCACGGACGCCTTCACAG-3′ with the following amplification cycles: heating at 95 °C for 15 min, followed by 36 cycles of denaturation at 94 °C for 15 s, annealing at 55 °C for 30 s and elongation at 72 °C for 30 s [46].

### 4.11. Statistical Analysis of the In Vivo Study

Worm burden, peritoneal mf counts, overnight mf sheds and qPCR data were analyzed using a Kruskal–Wallis test followed by Dunn’s multiple comparison. To determine significant differences in embryograms, a two-way ANOVA was conducted with a Dunnett’s multiple comparisons test, and significance levels were determined based on comparisons with vehicle treated animals. All statistical analyses were determined using Prism 8 version 8.2.0(272).

### 4.12. Transmission Electron Microscopy of Ov L5 (In Vitro) and Brugia pahangi Adult Females (In Vivo)

Ov L5 treated with 10 µM THP in vitro for 21 days and untreated worms were prepared for ultrastructural analysis by transmission electron microscopy (TEM) as previously described [46]. Briefly, worms were fixed in 2.5% glutaraldehyde and 2% paraformaldehyde in 0.1 M sodium cacodylate buffer and then stored at 4 °C. Fixed worms were washed 3 times in the buffer only and then post-fixed in 2% osmium tetroxide for 1 h and washed 3 times in buffer again. The fixed worms were dehydrated by immersion in an ethanol series, infiltrated with propylene oxide, embedded in epon 812 resin, and then incubated in an oven at 60 °C for 48 h to polymerize. An RMC MTX ultramicrotome with a Diatome diamond knife was used to cut ultrathin sections, which were stained using Uranyless (EMS, USA) and Reynold’s lead citrate. Images of samples were obtained using a FEI Tecnai 12 Spirit transmission electron microscope at 80 kV.

*B*. *pahangi* adult females recovered from gerbils treated with 1 mg/kg THP and analog 06 were prepared as above except worms were cut into approximately 5 mm long pieces taken from the center of the worm. Pieces were washed for one hour in 0.1 M sodium cacodylate followed by post-fixation in 1% osmium tetroxide for 1 h and again washed in the same buffer for another hour. Sample pieces were dehydrated using an increasing concentration of ethanol and resin (Durcupan, by Fluka) and transitioned using propylene oxide. Samples were left overnight in pure resin at 4 °C. Samples pieces were embedded using Beem capsules (Electron Microscopy Sciences) and cured at 60 °C overnight. All reagents were purchased from Electron Microscopy Sciences.

Blocks were trimmed and sectioned using a diamond knife on a Sorvall MT-6000 ultramicrotome, collected on formvar coated 1 mm slot grids and contrasted using uranyl acetate and lead citrate. Grids were imaged on a Philips CM100 transmission electron microscope at 80 kV and images were collected using a Gatan Orius imaging system.

## 5. Conclusions

PVP and its two analogs, THP and 06, were highly potent against filarial worms across life history stages and species in in vitro worm assays with IC_50_s in the sub-nanomolar to sub-micromolar range. These compounds impaired female worm fecundity and embryogenesis in in vivo studies. Because transmission of filariasis and the clinical pathology of river blindness are driven by the presence of microfilariae in the human host, a macrofilaricidal drug that confers long-term sterility of female worms is also viewed as a viable therapeutic approach. PVP, THP and 06 were also potent in preventing the molting of *Onchocerca volvulus* infective third-stage larvae, which also positions them for potential use as chemoprophylactic drugs in preventing larvae from developing into the adult stages. In conclusion, PVP and its analogs show promise as new leads for further drug development against filarial worm infections both for chemoprophylactic use and macrofilaricidal therapy.

## Figures and Tables

**Table 1:** Results of the in vitro whole-worm assay.

Compound	*Brugia* Adult Females Inhibition of Motility Day 3 @ 1 µM	*Brugia* Adult Females Inhibition of Motility Day 3 IC_50_ (µM)	*O*. *volvulus* L5 Inhibition of Motility Up to Day 36	*O*. *volvulus* L5 Inhibition of Motility IC_50_ (µM)	*O*. *volvulus* L3 Inhibition of Molting Day 6 IC_50_ (µM)	*O*. *ochengi* Adult Males Inhibition of Motility Day 5 IC_50_ (µM)	*O*. *ochengi* Adult Females Inhibition of Survival Day 7 IC_50_ (µM)	*O*. *ochengi* Mf Inhibition of Motility Day 5 @ 10 µM	*Loa loa* Mf Inhibition of Motility Day 5 @ 10 µM
01	99%	0.008	94% on Day 36 @ 1 µM	nd	nd	0.96	0.79	100%	100%
02	83%	0.001	90% on Day 16 @ 1 µM	nd	nd	3.08	0.70	100%	100%
03	3%	nd	97% on Day 28 @ 10 µM	nd	0.14	1.54	5.18	0%	13%
04	26%	nd	nd	nd	nd	* 100% @ 10 µM	nd	0%	38%
05	81%	0.405	92% on Day 36 @ 1 µM	nd	nd	* 86% @ 10 µM	nd	0%	38%
06	71%	0.733	94% on Day 36 @ 1 µM	0.22 µM on Day 21	0.46	2.75	2.99	46%	88%
THP	100%	0.003	68% on Day 36 @ 1 µM	<1.0 µM on Day 21	0.19	0.06	0.61	100%	100%
PVP	82%	0.0003	100% on Day 14 @ 1 µM	<0.3 µM on Day 21	0.03	0.11	0.05	100% and ** IC_50_ = 0.3 µM	100%

nd = not done; * Assays conducted at only a single concentration (10 µM); no further testing done; ** IC_50_ was also conducted with *O*. *ochengi* mf.

**Table 2:** In vitro ADME results. Analog 06 showed improved retention in the gerbil liver microsome assay as compared to its parent compound and the other analogs. PVP and the analogs except for 06 showed rapid clearance from microsomes, indicating that it could be rapidly metabolized in the liver.

Compound	Gerbil Liver Microsome T_1/2_ (min)	CL_int_ (µL/min/mg Protein)	Mean Papp_A-B_ (×10^−6^ cm/s)	Mean Papp_B-A_ (×10^−6^ cm/s)	MDCK Efflux Ratio B-A/A-B	MDCK Recovery Rate (%)
01	2.3	610.8	0.1	61.3	531.9	77
02	2.6	541.8	0.1	51.2	736.7	67
03	4.9	280.2	1.3	9.1	7.2	104
05	7.7	179.88	0.7	6.8	9.3	96
06	1484.9	0.933	0.5	12.1	23.7	94
THP	14.6	95.14	<0.25	0.6	>2.5	15
PVP	5.3	261.6	<0.07	6.6	>94.6	69

**Table 3:** Treatment with PVP reduces adult worm burden and mf levels in the peritoneal cavity. The mean number of adult worms and mf from animals given 1 mg/kg PVP IP was reduced by 20- and 100-fold, respectively, compared to vehicle treated animals. Means ± SEM (in parentheses) are shown.

	Dosage of 1 mg/kg			
	Vehicle	THP	06	PVP
Number of animals	8	8	8	7
Adult worms per animal	18.5 (8.2)	21.9 (7.6)	31.3 (11.8)	**0.9 (0.5)**
Female worms per animal	8.5 (3.9)	10.4 (4.2)	15.9 (5.7)	**0.1 (0.1)**
Male worms per animal	10 (4.3)	11.5 (3.7)	15.4 (6.1)	**0.7 (0.4)**
Mf per animal	1,271,443 (1,092,551)	652,500 (326,014)	915,000 (263,235)	**10,429**
				**(5822)**

## Data Availability

Not applicable.

## Supplementary Materials

The following are available online at https://www.mdpi.com/article/10.3390/ph15020189/s1, Figure S1: Molting assay; Figure S2: *Wolbachia* titers from female worms; Table S1: MTT assays; Table S2: Embryogram analyses.
