# Supplementary material for: Pyrvinium Pamoate and Structural Analogs Are Early Macrofilaricide Leads
Source: Pharmaceuticals (Basel). 2022 Feb 2;15(2):189. doi: 10.3390/ph15020189 (PMC8880385; doi:10.3390/ph15020189)
Supplement: Supplementary file 1 [file pharmaceuticals-15-00189-s001.zip › pharmaceuticals-1452218-supplementary.pdf]

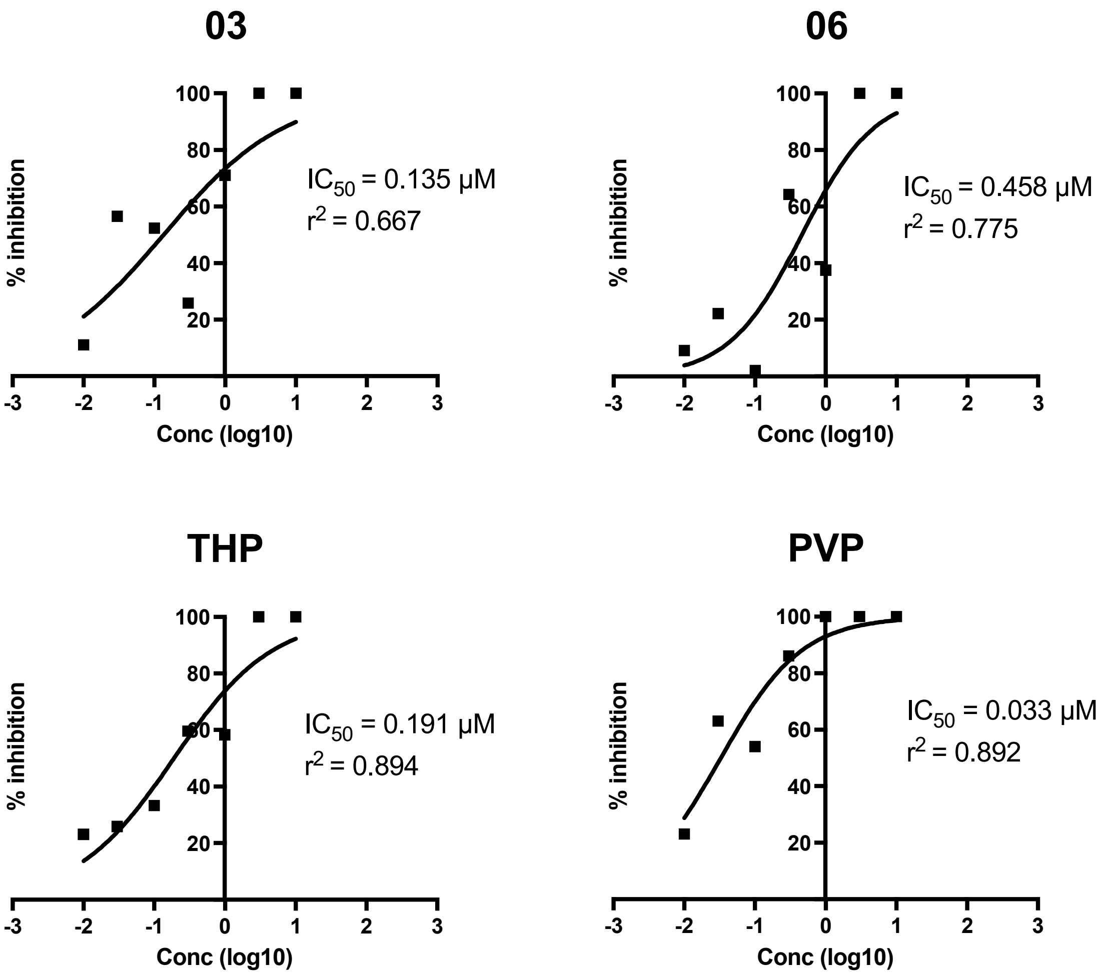

Supporting Information Figure S1. Molting assay. The number of L3 that molted to L4 *in vitro* was counted for each treatment group and controls. The IC_{50}s were calculated using the percent inhibition of molting compared to the proportion of L3 under control conditions that molted to L4.

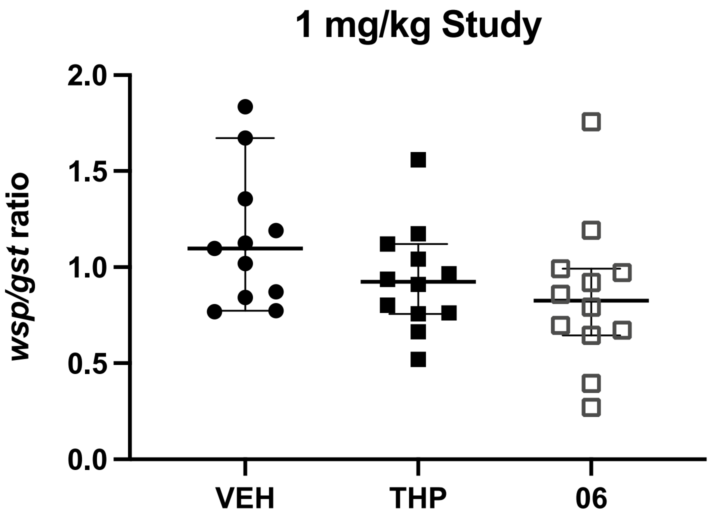

Supporting Information Figure S2. *Wolbachia* titers from *B. pahangi* female worms recovered at necropsy. There was no reduction in *Wolbachia* titers in female worms from any treatment group as measured by qPCR.

**Compound**	***O. volvulus* L5 (Ov L5) % inhibition of motility Day 36 @ 1 uM (worm assay)**	***O. volvulus* L5 (Ov L5) % inhibition of viability Day 37 (MTT assay)**
01	94	87
02	*90 (day 16)	nd
05	92	76
06	94	88
THP	68	52
PVP	**100 (day 14)	100
DMSO	0	0

Supporting Information Table S1. Comparison of the inhibition of Ov L5 worm motility and worm viability as measured by the MTT assay were well-correlated; *assay with 02 was stopped on day 16 due to contamination and MTT was not done (nd); **all worms treated with PVP were dead by day 14.

Stages of development	Vehicle	THP	06	PVP
Eggs	56.0 ± 6.3	43.5 ± 6.9	34.3 ± 6.4 **(P<0.05)**	*
Embryos	14.6 ± 1.7	10.9 ± 2.0	9.6 ± 1.9	*
Pre-mf	8.9 ± 1.6	12.2 ± 3.0	10.0 ± 2.1	*
Stretched mf	13.8 ± 3.5	6.7 ± 1.5	7.6 ± 1.6	*
Deformed	6.7 ± 4.4	26.8 ± 11.0 **(P<0.05)**	38.5 ± 11.0 **(P<0.001)**	*

Supporting Information Table S2: Embryogram analyses. Mean counts of each developmental stage ± SEM shown above. 200 events were counted for each worm. *Only one female worm was recovered from the PVP group so no embryogram analysis was conducted.
